# The Reproducibility of an Inferred Tree and the Diploidization of Gene Segregation after Genome Duplication

**DOI:** 10.1093/gbe/evz272

**Published:** 2020-01-17

**Authors:** Yukako Katsura, Masatoshi Nei

**Affiliations:** 1 Primate Research Institute, Kyoto University, Japan; 2 Department of Biology, Institute of Molecular Evolutionary Genetics, Pennsylvania State University; 3 Department of Biology, Institute for Genomics and Evolutionary Medicine, Temple University

**Keywords:** phylogenetic trees, genome duplication, reliability, stability, reproducibility, diploidization, gene segregation, computer program RESTA, MHC class II α, and β, chain gene families

## Abstract

We previously introduced a numerical quantity called the stability (Ps) of an inferred tree and showed that for the tree to be reliable this stability as well as the reliability of the tree, which is usually computed as the bootstrap probability (Pb), must be high. However, if genome duplication occurs in a species, a gene family of the genome also duplicates, and for this reason alone some Ps values can be high in a tree of the duplicated gene families. In addition, the topology of the duplicated gene family can be similar to that of the original gene family if such gene families are identifiable. After genome duplication, however, the gene families are often partially deleted or partially duplicated, and the duplicated gene family may not show the same topology as that of the original family. It is therefore necessary to compute the similarity of the topologies of the duplicated and the original gene families. In this paper, we introduce another quantity called the reproducibility (Pr) for measuring the similarity of the two gene families. To show how to compute the Pr values, we first compute the Pb and Ps values for each of the MHC class II α and β chain gene families, which were apparently generated by genome duplication. We then compute the Pr values for the MHC class II α and β chain gene families. The Pr values for the α and β chain gene families are now low, and this suggests that the diploidization of gene segregation has occurred after the genome duplication. Currently higher animals, defined as animals with complex phenotypic characters, generally have a higher genome size, and this increase in genome size appears to have been caused by genome duplication and diploidization of gene segregation after genome duplication.

## Introduction

**Fig. 1.**
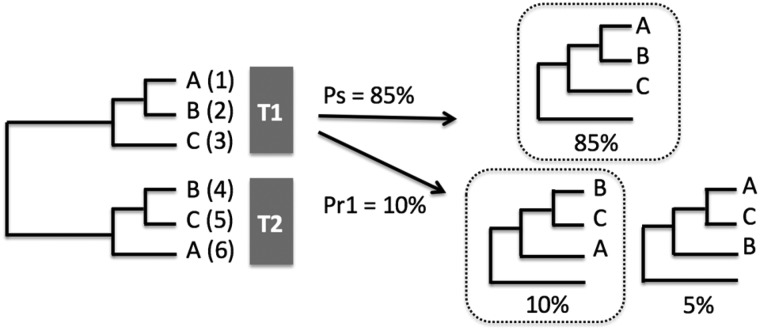
—An example of computation of the Ps and Pr values. We assume that the original inferred tree of a gene family is composed of DNA or protein sequences (1), (2), and (3): Its topology is ((A,B), C) called T1 (Topology 1), and the tree of duplicated gene family is composed of sequences (4), (5) and (6): Its topology is ((B,C), A) called T2 (Topology 2). The T1 represents three possible subtree topologies, which is expressed as ((A,B), C), ((B,C), A), and ((A,C), B), and one of the close outgroups is used. The number of bootstrap replications used for each subtree topology is assumed to be 1,000. The probability of obtaining a subtree topology is shown as a percentage for each of the three subtree topologies (85%, 10%, and 5%). The Ps value is the bootstrap value for the tree topology showing the highest bootstrap probability among the three subtree topologies (85%), and in the present case we have assumed that it is the subtree topology having ((A,B), C). In this study, we compute the reproducibility of topologies between the original and the duplicated gene families. The reproducibility value (Pr) is the average of the bootstrap probability (Pr1) showing T2 among the replicates of the subtree of T1 and the bootstrap probability (Pr2) showing T1 among the replications of the original tree of T2. This figure shows that Pr1 is 10%, and Pr2 is not shown.

In the previous paper, we introduced a numerical quantity called the stability for measuring the accuracy of an inferred tree from empirical data (Katsura et al. 2017). This stability refers to the bootstrap probability of the subtree to be defined (Ps), and we have shown that for the inferred tree to be reliable both the Ps values and the usual bootstrap values (Pb) must be high (Katsura et al. 2017). For the people who are not well acquainted with the statistical method used, we present the essence of Ps and Pr computation in figure 1. In this paper, we introduce another quantity called the reproducibility (Pr) of the inferred tree when two homologous gene families exist. The Pr value refers to the similarity of the topologies of the two homologous gene families. When genome duplication occurs in a species, a gene family of the genome should also duplicate. If the duplicated gene family shows the same topology as that of the original family, Pr is 100%, whereas if the two topologies are entirely different, it is 0%. A low value of reproducibility indicates that the topologies of the ancestral or duplicated gene families have been altered considerably by the postduplication events such as the partial gene deletion or partial gene duplication (Scannell et al. 2006; Nei 2013, pp. 139–140).

In this paper, we first explain the computational principle in an example case. We then use the α and β chain genes of the major histocompatibility complex (MHC) class II genes as the numerical example, because the α and β chain genes were apparently generated by genome duplication (see Nei and Hughes 1991; Katsura et al. 2017). In the class II region of the MHC, the α and β chains in the mammal immunoglobulins are encoded by separate genes in the DP, DQ, and DR gene regions. The genes in the DP, DQ, and DR gene regions are responsible for presenting the peptides derived from extracellular pathogens to cytotoxic T cell. We do not consider the DM and DO regions because they do not present antigens (Ting and Trowsdale 2002). In humans, the DP and DQ regions possess one functional gene for each of the α and β chains, and the DR region contains one copy of the functional α chain gene and two functional copies of the β chain gene (β1 and β3 genes) (Nei and Hughes 1991; Ting and Trowsdale 2002). In this study, we used the α1 and β1 genes in all of the DP, DQ, and DR gene regions (Nei and Hughes 1991). The α and β chain genes of the DP region (DPA and DPB genes, respectively) are absent from many currently known mammalian genomes except in primates, rodents, and elephants, and the DPB genes are pseudogenes in rodents (Katsura et al. 2017). In this paper, we consider the primate MHC class II genes (DNA sequences) only to avoid the problem of the gene deletion or gene duplication that has occurred in nonprimate species. We also compare the gene sequences from the same number of species for each of the DP, DQ, and DR gene regions. We used the nucleotide sequences from a total of nine primate species in which the entire genome sequence is available. These species included three hominoids, three Old World monkeys, two New World monkeys, and one Prosimian. The nine species used here are human (*Homo sapiens*), chimpanzee (*Pan troglodytes*), gorilla (*Gorilla gorilla*), rhesus macaque (*Macaca mulatta*), drill (*Mandrillus leucophaeus*), black snub-nosed monkey (*Rhinopithecus bieti*), common marmoset (*Callithrix jacchus*), Ma’s night monkey (*Aotus nancymaae*), and gray mouse lemur (*Microcebus murinus*). The phylogenetic trees of these species were previously studied by Perelman et al. (2011), Springer et al. (2012), and Perez et al. (2013); and the topology of our tree was generally in agreement with that of their trees. 

## Results

**Fig. 2.**
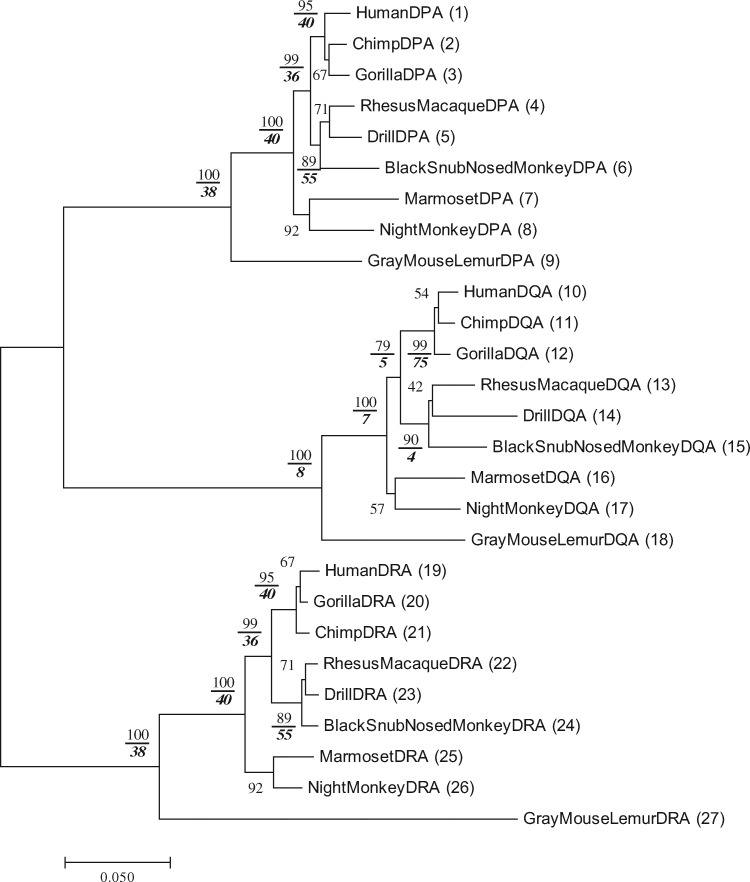
—Pb and Ps values of the phylogenetic tree for 27 MHC class II DPA, DQA, and DRA genes in 9 primates. The phylogenetic tree obtained by the NJp method (Saitou and Nei 1987; Yoshida and Nei 2016). The number of nucleotides used was 939 bp per sequence. The Pb value is shown for each interior branch, and the number of bootstrap replications used was 1,000. The Ps value was also obtained by the 1,000 replications for each gene family using the RESTA program (Katsura et al. 2017) and is shown as a bold and italic number below the Pb value for each relevant interior branch. The aligned sequences are in Supplementary Material online.

Figure 2 shows the phylogenetic tree of MHC class II α chain genes. The Pb and Ps values for each relevant interior branch are presented in figure 2. The Ps values are given by an italic and bold letter below the Pb value for each relevant interior branch. We have not shown the Ps values when there are only two sequences in the subtree for the reason mentioned in Katsura et al. (2017). The Pb and Ps values were computed by the computer program RESTA as mentioned in Katsura et al. (2017). The Ps values in figure 2 are generally similar to the Pb values, but occasionally the Ps value is drastically lower than the Pb value.

**Fig. 3.**
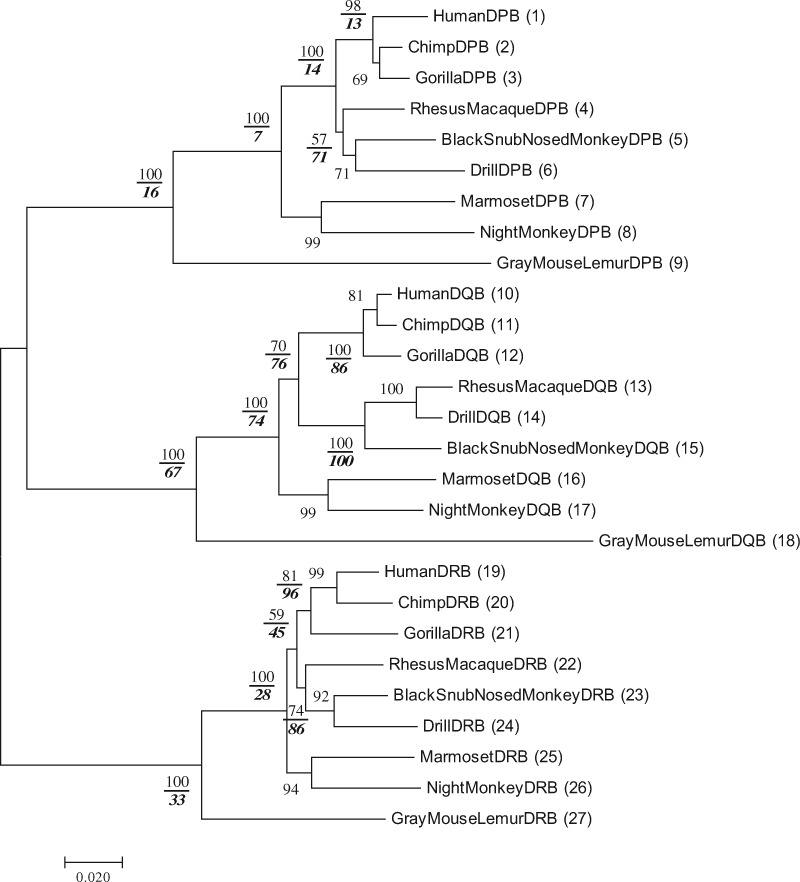
—Pb and Ps values of the phylogenetic tree for 27 MHC class II DPB, DQB and DRB genes in 9 primates. The Ps value is shown in a bold and italic number below the Pb value for each relevant interior branch. The number of nucleotides used was 927 bp per sequence.

Figure 2 shows that the topology of the tree is generally consistent with that of the primate trees obtained in the previous studies (Perelman et al. 2011; Springer et al. 2012). The topology of DQA genes (sequences 10-18) is identical with a correct topology supported by the previous studies (Perelman et al. 2011; Springer et al. 2012). The topologies of the hominoids in DPA and DRA genes (sequences 1-3 and sequences 19-21) are not identical with that of DQA genes (sequences 10-12). The topology of the hominoid cluster is often different from that of the previous studies, although the Pb values of the hominoid cluster are always high. The Ps value of the hominoid cluster in the DPA gene is 40%, and this low Ps value suggests that the subtree of the hominoid cluster is unreliable in the DPA gene. The instability of the tree can be detected by computing both Pb and Ps values, and this conclusion is similar to that of figure 3. Figure 3 shows the Pb and Ps values for an interior branch of MHC class II β genes. The Ps values of primate MHC class II β genes are mostly lower than the Pb values. This was true also with our previous results obtained by using mammalian MHC class II β genes (Katsura et al. 2017).

We now compute the Pr values for the trees of three groups of the α and β chain genes, that is, the DP, DQ, and DR family genes in figures 2 and 3. Let us explain how to compute the Pr values by considering the subtree for the DP gene region genes. The Pr value for this subtree is obtained by computing the bootstrap probability of obtaining the topology of the DPA subtree in the subtree of DPB genes when one of the close outgroups is used. Let us designate the topology of DPA genes in figure 2 by T1, and the topology of DPB genes in figure 3 by T2. Here we have two ways of computing the reproducibility for a given topology of DP genes. One way of computation is to consider the reproducibility of topology T2 of DPB genes in the subtree for DPA genes. In this case, we compute the Pr value by considering how often topology T2 is obtained by bootstrapping the subtree of DPA genes with the closest outgroup. Let us call this reproducibility Pr1. The Pr value is also computable by considering the subtree of DPB genes for obtaining the topology T1 with the closest outgroup. We designate this bootstrap probability by Pr2. The Pr value is then obtained by the average value of Pr1 and Pr2. Using this way of computation, we also calculate Pr1, Pr2, and Pr for DQ and DR genes. For this computation, the program RESTA can be useful.

The Pr1, Pr2, and Pr thus obtained are presented in table 1*A*. The topologies of the α and β chains are slightly different in DR and DP genes. The Pr values for DR and DP genes therefore become low (0%). The topologies of the genes are not reproducible between the α and β chains. On the other hand, the Pr for DQ genes between the α and β chains is 37.4% and relatively higher than that for DR and DP genes because the topologies of DQA and DQB genes are exactly the same. However, the Pr for DQ genes was not 100% but the Pr2 is 66.8% and Pr1 is 8.0%. This relatively low Pr1 value is caused by the instability of the subtree in DQA gene. The Pb is 100% for the subtree of DQA genes (sequences 10-18), but the Ps is only 8% for the subtree (fig. 2). The low Ps value makes it hard for the subtrees in DQA genes to reproduce the topologies of DQB genes. Our results show that the Pr values become high if the topologies of the duplicated gene families are exactly the same. Furthermore, if trees of the duplicated gene families are highly reliable in terms of both Ps and Pb values, Pr values can become higher.

**Table 1:** The Pr1, Pr2, and Pr Values for the Phylogenetic Tree

(A) The Pr1, Pr2, and Pr Values Between the α and β Chain Genes for the Tree of DP, DQ, and DR Genes Are Shown in Percentage, Respectively			
	Pr1	Pr2	Pr
DPA–DPB	0.1	1.3	0.7
DQA–DQB	8.0	66.8	37.4
DRA–DRB	0.5	0.0	0.3

**(B) The Pr1, Pr2, and Pr Values for the DP–DQ, DQ–DR, and DR–DP Comparison for the α or β Chain Genes, Respectively**			

DPA–DQA	0.5	13.0	6.8
DPA–DQB	12.7	0.0	6.4
DPB–DQA	3.4	2.0	2.7
DPB–DQB	0.0	0.0	0.0
DQA–DRA	10.7	1.7	6.2
DQA–DRB	42.7	1.4	22.1
DQB–DRA	11.2	6.7	9.0
DQB–DRB	2.0	1.4	1.7
DRA–DPA	1.1	1.6	1.4
DRA–DPB	0.0	15.7	7.9
DRB–DPA	0.1	0.0	0.1
DRB–DPB	5.7	0.0	2.9

## Discussion

We quantified the reproducibility (Pr) between the α and β chain genes of MHC class II genes. This could be done because MHC class II genes function as heterodimers of α and β chains, and we could identify the α and β chain genes as the duplicated gene families. Grimholt (2016), who studied the immune system in fish extensively, believes that the class II immune system originated in teleost fish about 250 Ma. Figures 2 and 3 show that the gene duplication events in the DP, DQ, and DR gene regions occurred more recently, so that all of them include the α and β chain genes. It is believed that the duplication of MHC class II α and β chain genes originated from the whole genome duplication event in the teleost ancestor (Grimholt 2016). Soon after a genome was duplicated, the topology of the duplicated gene families can be identical with that of the ancestral gene family. Then, Pr values between the duplicated gene families would be high. Actually, however, the Pr values between the α and β chain genes are low in DP and DR gene regions since the topologies of the α and β gene families are different.

We computed the Pr value for all pairs of the DP–DQ, DP–DR, and DQ–DR (table 1*B*). The Pr values are low (0.0-2.7%) in three pairs of the genes (DPB–DQA, DPB–DQB, and DRB–DPA), and the topologies of these duplicated genes are different at two interior branches for each pair (table 1*B*). Although the Pr values are very rarely 0% and low (1.4-22.1) in the other nine pairs of the gene families showing the topological difference at one interior branch (table 1*B*). The Pr values became lower depending on the difference of topologies in the trees of the duplicated and original gene families, but the Pr values are relatively low in any pair.

Britten and Davidson (1969) showed that higher animals generally have a higher genome size, and this continuous increase of genome size has made it possible that higher organisms have complex phenotypic characters. Nei (1969) suggested that this continuous increase in genome size was possible if the majority of the increase occurred by means of unequal crossing over. However, the increase in genome size was also possible if the gene segregation of autotetraploids became diploidized (Ohno 1970, pp. 101–104). Scannell et al. (2006) showed that this type of diploidization indeed occurred in yeast. In this paper, the duplicated α and β chain genes maintained similar functions, and therefore it was easy to identify the α and β chains as the duplicate genes. Following the accumulation of mutations, insertions, and deletions of the genes, the sequences of the duplicated genes diverged from those of the original genes (Nei 1969, 2013). The low Pr values of the primate α and β chain genes support the occurrence of diploidization after genome duplication. The Pr computation using duplicate genes can quantify the extent of diploidization in tetraploids in addition to the topological similarity of the gene families. In this study, we calculated the Pr for MHC class II α and β chain genes, but the computation of the Pr can be done in any duplicated gene families (fig. 1). 

## Acknowledgments

This work was supported by JSPS KAKENHI Grant Number 18K14766.

## Supplementary Material

Supplementary data are available at *Genome Biology and Evolution* online.

## Supplementary Material

evz272_Supplementary_Data
